# Supplementary material for: Identification of Withaferin A as a Potential Candidate for Anti-Cancer Therapy in Non-Small Cell Lung Cancer
Source: Cancers (Basel). 2019 Jul 17;11(7):1003. doi: 10.3390/cancers11071003 (PMC6678286; doi:10.3390/cancers11071003)
Supplement: Supplementary file 1 [file cancers-11-01003-s001.pdf]

Supplementary materials

Identification of Withaferin A as a Potential Candidate for Anti-Cancer Therapy in Non-Small Cell Lung Cancer

Jade H.-M. Hsu, Peter M.-H. Chang, Tai-Shan Cheng, Yu-Lun Kuo, Alexander T.-H. Wu, Tran Thu Ha, Yun-Hsuan Yang, Jing-Ming Chen, Yu-Chen Tsai, Yeh-Shiu Chu, Tse Hung Huang, Chi-Ying F. Huang and Jin-Mei Lai

Table S1. Drug list of fifty-seven drugs from bioinformatics selection.

Top100, LOU, SAM	ratio	Appearance more than 20%	IC50 (μM)
3	100%	Emetine	<0.1
3	100%	Withaferin A	<1
3	100%	Trichostatin A	<1
3	100%	Tanespimycin	<1
3	100%	Sanguinarine	<1
3	100%	Thioguanosine	<5
3	100%	Vorinostat	>5
3	100%	Chlorpromazine	>5
3	100%	Bepridil	>5
3	100%	Amiodarone	>5
3	100%	Procaine	>10
3	100%	Tyloxapol	>10
3	100%	Trioxysalen	>10
3	100%	Trifluoperazine	>10
3	100%	Trazodone	>10
3	100%	Sulfametoxydiazine	>10
3	100%	Phthalylsulfathiazole	>10
3	100%	Phenoxybenzamine	>10
3	100%	Morantel	>10
3	100%	Milrinone	>10
3	100%	Methylergometrine	>10
3	100%	Medrysone	>10
3	100%	Luteolin	>10
3	100%	Levonorgestrel	>10
3	100%	Gliclazide	>10
3	100%	Ginkgolide A	>10
3	100%	Fenoprofen	>10
3	100%	Eucatropine	>10
3	100%	Diltiazem	>10
3	100%	Clioquinol	>10
3	100%	Apigenin	>10
3	100%	Evoxine	>10
3	100%	Leucomisine	>10
3	100%	DL-thiorphan	>10
3	100%	8-azaguanine	>10
3	100%	15-delta prostaglandin J2	>10
3	100%	Tranylcypromine	ND
3	100%	Sulfafurazole	ND
3	100%	Resveratrol	ND
3	100%	Quinostatin	ND
3	100%	Puromycin	ND
3	100%	Promethazine	ND
3	100%	Piperidolate	ND
3	100%	Lisinopril	ND
3	100%	Fluphenazine	ND
3	100%	Ethotoin	ND

3	100%	Clomipramine	ND
3	100%	Cinchocaine	ND
3	100%	Chlorzoxazone	ND
3	100%	Cephaeline	ND
3	100%	Cefalexin	ND
3	100%	Apramycin	ND
3	100%	Alvespimycin	ND
3	100%	Y-27632	ND
3	100%	LY-294002	ND
3	100%	5224221	ND
3	100%	4,5-dianilinophthalimide	ND

Fifty-seven drugs from all three selection criteria (Top100, LOU, and SAM); 36 drugs were analyzed in in vitro cytotoxic experiments for determining IC50 values in our laboratory. LOU: Leave-one out; ND: not determined.

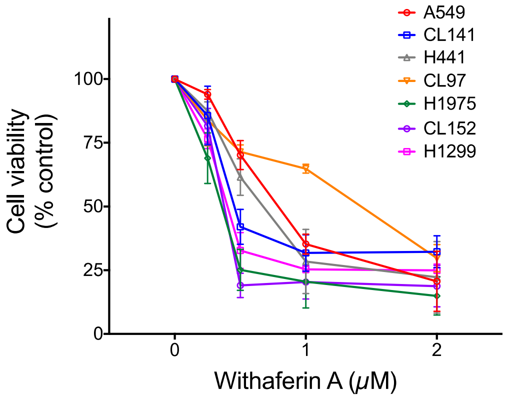

Figure S1. Cytotoxicity effect of WA on various lung cancer cell lines. Representative cell viability curves of the IC_{50} of WA after 48-h treatment in A549, CL141, H441, CL97, H1975, CL152, and H299 cells (*n* = 3).

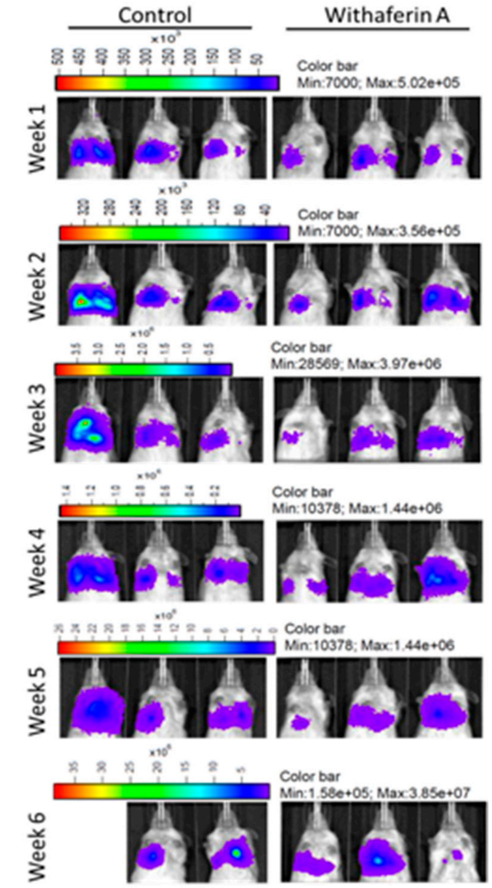

Figure S2. Image of WA-treated mice. Three of each group representative bioluminescence images of H441 lung adenocarcinoma-bearing mice. WA treatment suppressed tumorigenesis, as indicated by the lower bioluminescence intensity. Mice were observed over six weeks.
